# Clinical validity of dried blood spot assay for the measurement of functional C1 inhibitor in angioedema due to C1 inhibitor deficiency

**DOI:** 10.1016/j.jacig.2025.100401

**Published:** 2025-01-07

**Authors:** Jonathan A. Bernstein, Jie Cheng, Thomas Pisani, Dan Sexton, Rachel E. Whitaker, Daniel Nova Estepan, Neil Inhaber

**Affiliations:** a Division of Rheumatology, Allergy, and Immunology, University of Cincinnati College of Medicine and Bernstein Clinical Research Center, Cincinnati, Ohio; b Takeda Development Center Americas, Inc, Lexington, Mass

**Keywords:** Assay, diagnostics, functional C1 inhibitor, hereditary angioedema

## Abstract

**Background:**

Functional C1 inhibitor (fC1INH) is a key biomarker for the diagnosis of hereditary angioedema due to C1 inhibitor (C1INH) deficiency. A novel fC1INH assay from dried blood spot (DBS) reduces practical fC1INH testing limitations versus conventional fC1INH assays, but its sensitivity and specificity have not yet been characterized.

**Objective:**

We sought to assess the sensitivity and specificity of fC1INH DBS assay and conventional fC1INH ELISA and chromogenic assay.

**Methods:**

fC1INH DBS assay was performed in samples from 30 patients with previously diagnosed recurrent angioedema due to C1INH deficiency and from 100 healthy controls. Whole blood samples were spotted onto blotting paper and dried for 3 hours or longer, and then fC1INH from DBS was measured through its C1s protease inhibitory activity using liquid chromatography tandem mass spectrometry. fC1INH ELISA and chromogenic assays were performed by reanalyzing a subset of 29 patients and 50 healthy controls. Sensitivity and specificity were evaluated using a negative sample mean − 1.96 SD cutoff.

**Results:**

fC1INH DBS assay had a sensitivity of 0.93, a specificity of 0.97, and area under the curve of the receiver-operating characteristic curve of 0.996 (95% CI, 0.989-1.000). In the subset, DBS and chromogenic assay had similar sensitivity (DBS, 1.00; chromogenic assay, 0.97) and specificity (DBS, 0.94; chromogenic assay, 1.00); ELISA sensitivity was lower (0.62) and specificity similar (1.00).

**Conclusions:**

fC1INH DBS assay had similar sensitivity and specificity when contrasted with fC1INH chromogenic assay. Because of its easier sample collection and logistic benefits, fC1INH DBS assay may allow more accessible hereditary angioedema diagnostic testing, especially in underserved regions.

Hereditary angioedema (HAE) is a rare genetic disease that clinically presents as recurrent swelling attacks of skin and subcutaneous tissues.^1^ HAE is most frequently caused by mutations in the *SERPING1* gene coding for C1 inhibitor (C1INH), resulting in quantitative C1INH deficiency (HAE Type I) or C1INH dysfunction (HAE Type II), collectively known as HAE due to C1INH deficiency (HAE-C1INH).^2^^,^^3^ HAE-C1INH is estimated to affect 1 in 50,000 individuals globally, with no known differences in incidence on the basis of sex, race, or ethnicity.^3^^,^^4^ HAE clinically manifests as recurrent attacks of subcutaneous or submucosal swelling, which are generally unpredictable and may affect the face, extremities, gastrointestinal tract, and genitalia.^5^ HAE attacks that affect the upper airway/larynx affect up to 50% of patients with HAE and pose a notable risk of mortality due to asphyxiation, especially in undiagnosed patients with HAE.^6^, ^7^, ^8^

HAE can be clinically suspected in patients presenting with a history of recurring swelling attacks of the skin (extremities, face, and/or genitalia) and/or gastrointestinal tract (abdominal symptoms and/or laryngeal edema).^3^ However, the clinical presentation of HAE-C1INH can be similar to other conditions of angioedema without urticaria, including HAE with normal C1INH, acquired angioedema due to C1INH deficiency, acquired angiotensin-converting enzyme inhibitor–associated angioedema, and nonhistaminergic idiopathic angioedema; allergic and idiopathic histamine-mediated angioedema may sometimes also present with angioedema but without urticaria.^9^^,^^10^ Therefore, laboratory investigations are required to fully establish the diagnosis of HAE-C1INH.^3^^,^^11^^,^^12^

In patients with HAE Type I, the antigenic C1INH level, C1INH function, and antigenic C4 level are reduced, whereas in patients with HAE Type II, the antigenic C1INH level is normal or elevated but C1INH function and antigenic C4 level are reduced.^13^ The antigenic C4 level is reduced in most but not all patients with HAE-C1INH, leading to low sensitivity of C4 testing for diagnosis of HAE-C1INH.^14^ Accordingly, current guidelines for HAE management do not recommend C4 testing alone to establish a diagnosis of HAE-C1INH.^3^^,^^11^^,^^12^ Instead, diagnostic workup for HAE-C1INH as recommended by current guidelines optimally includes testing for antigenic C4 level, antigenic C1INH level, and C1INH function,^3^^,^^11^^,^^12^ and repeated testing is recommended to confirm the diagnosis.^3^^,^^11^ Furthermore, diagnosis of acquired angioedema due to C1INH deficiency may need to be excluded in patients with negative family history and symptom onset after the age of 30 years; antigenic C4, antigenic C1INH, and functional C1INH (fC1INH) findings of this condition are consistent with HAE Type I.^3^ fC1INH is markedly reduced in both HAE Type I and HAE Type II; therefore, its measurement is important for diagnosis of HAE-C1INH as the most straightforward assessment.^3^ However, fC1INH assessment may be limited by the necessity to have skilled laboratories for correct processing and interpretation of commercially available fC1INH testing kit results.^3^^,^^13^ Antigenic C4 level, antigenic C1INH level, and fC1INH can be classified as complement cascade biomarkers of HAE.^15^ Although not currently used in routine practice, bradykinin-forming cascade proteins (activated factor XII, cleaved high-molecular-weight kininogen, and plasma kallikrein) have also been described as potential biomarkers for HAE.^15^ Activated factor XII cleaves prekallilkrein to generate plasma kallikrein, which, in turn, cleaves high-molecular-weight kininogen, leading to bradykinin release.^16^ As an illustration, inhibition of active plasma kallikrein with lanadelumab leads to decreasing levels of cleaved high-molecular-weight kininogen^17^ and, correspondingly, decreasing generation of bradykinin.

In clinical practice, 2 types of diagnostic tests for the measurement of fC1INH are generally available: fC1INH ELISA and fC1INH colorimetric (chromogenic) assay.^13^ Both of these methods require blood sample collection, serum/plasma separation by centrifugation as soon as possible after blood collection, and storage of serum/plasma samples in a refrigerator (for up to 2 days) or freezer at or below −20°C unless processed immediately.^13^^,^^18^^,^^19^ The equipment and expertise needed to correctly collect, process, and analyze samples for the conventional fC1INH diagnostic tests may contribute to the disparity in the availability of fC1INH testing. A recent survey in 10 low- and middle-income countries reported limited availability of essential diagnostics in most of the surveyed countries, highlighting the disparity in access to laboratory diagnostics overall.^20^ Accordingly, limited availability to test for C1INH function has been reported in multiple countries and regions, including the Asia Pacific region,^21^ Brazil,^22^ China,^23^ India,^24^ South Africa,^25^ and the Middle East region.^26^

Recently, a methodology for the fC1INH assay that uses dried blood spot (DBS) samples to measure fC1INH using liquid chromatography tandem mass spectrometry (LC-MS/MS) has been developed.^27^ DBS sampling is being increasingly used in various fields including newborn screening, drug development, and diagnosis of infectious diseases.^28^, ^29^, ^30^ DBS sample transportation at ambient conditions is simplified, enabling decentralized sampling with downstream analyses performed in a centralized laboratory. Sample collection in remote sites and analysis in a central laboratory may enable more widespread testing in rural locations as well as in underserved geographic regions.^29^

The performance of a diagnostic assay can be evaluated by assessing the analytical validity of the assay (ability to detect the presence or absence of analytes the assay is designed to detect), clinical validity (ability to correctly identify the patient’s clinical status), and clinical utility (benefits and risks resulting from the use of the assay).^31^^,^^32^ Area under curve (AUC) of the receiver-operating characteristic (ROC) curve, sensitivity, and specificity are all important measures to evaluate the clinical validity of an assay; AUC of the ROC curve allows the evaluation of the overall quality of a diagnostic test, whereas sensitivity and specificity allow distinguishing between false-positive and false-negative results.^33^^,^^34^ The analytical validity of the fC1INH LC-MS/MS assay from DBS has been reported previously,^27^ but the sensitivity and specificity of the fC1INH LC-MS/MS assay from DBS remain to be established. In this study, the sensitivity and specificity of the fC1INH LC-MS/MS assay were contrasted with those from 2 alternative fC1INH diagnostic tests: fC1INH ELISA and fC1INH chromogenic assay.

## Methods

### Study design and population

This study used a case-control design and included samples from patients previously diagnosed with recurrent angioedema due to C1INH deficiency as a part of their regular care unrelated to this study and from healthy subjects. Whole blood from 30 patients with recurrent angioedema due to C1INH deficiency was provided by a large allergy group in the Midwest United States with patients' written informed consent. Institutional review board approval was provided by the independent Advarra Institutional Review Board. Samples from patients with recurrent angioedema due to C1INH deficiency were collected during the time period from February 2019 to January 2020; patient selection used convenience sampling. Human whole blood from 100 apparently healthy subjects was purchased from BioIVT (Westbury, NY).

### Study assessments

The fC1INH LC-MS/MS assay from DBS was performed using a previously published method.^27^ Briefly, samples of whole blood from patients with recurrent angioedema due to C1INH deficiency were collected by drawing venous blood into EDTA-coated tubes. EDTA-treated whole blood samples from healthy subjects were obtained as described earlier. Samples of whole blood were collected on the DBS collection cards and dried for at least 3 hours; cards were then stored at −20°C in sealed biohazard bags with 1 desiccant per bag. DBS disks were cut using a 3.0-mm puncher and processed to extract a C1INH sample, which was subsequently bound with C1s protease. C1s protease substrate Z-Lys-Sbzl was then added to the sample, and an enzymatic reaction occurred between unbound C1s protease and Z-Lys-Sbzl. Finally, the reaction product (cbz-Lys) was measured using LC-MS/MS.^27^ The fC1INH LC-MS/MS assay from DBS was performed by Revvity Omics (previously PerkinElmer Genomics) in a blinded manner.

The fC1INH ELISA was performed by RayBiotech using a commercial Quidel kit (MicroVue C1-Inhibitor Plus Enzyme Immunoassay, catalog no. A037; Quidel, San Diego, Calif) by reanalyzing a subset of samples. EDTA-treated plasma samples were used for this analysis. ELISA measurements in the equivocal zone (41%-67% of the normal as per manufacturer’s instructions) were treated as negative in the main analysis.

The fC1INH chromogenic assay was performed by National Jewish Health Advanced Diagnostic Laboratories (Denver, Colo) using a commercial kit (Technochrom C1-INH; DiaPharma, West Chester Township, Ohio), by reanalyzing a subset of samples.

Because the fC1INH LC-MS/MS assay used more samples compared with the fC1INH ELISA and chromogenic assays, a sensitivity analysis was performed in which only the subset of samples reanalyzed using the fC1INH ELISA and chromogenic assays was considered when analyzing the performance of the fC1INH LC-MS/MS assay from DBS (DBS subset analysis). Another sensitivity analysis, in which ELISA measurements in the equivocal zone were treated as positive, was also performed (ELISA alternative analysis).

### Statistical analyses

The statistical analyses were performed in R version 4.2.0 (R Core Team, Vienna, Austria) using R package auRoc.^35^ The performances of the fC1INH DBS assay, the fC1INH chromogenic assay, and the fC1INH ELISA were evaluated by calculating AUC of the ROC curve. The 95% CI for the AUC of the ROC curve was also calculated. The reference interval was calculated as the healthy control mean ± 1.96 SD. Values higher than the lower end of the reference interval (the healthy control mean − 1.96 SD) were evaluated as normal (negative for recurrent angioedema due to C1INH deficiency [HAE or acquired angioedema due to C1INH deficiency]), and values lower than it were evaluated as positive for recurrent angioedema due to C1INH deficiency in all analyses except the ELISA main analysis, in which only the samples less than 41% of the normal as per manufacturer’s instructions were evaluated as positive for recurrent angioedema due to C1INH deficiency. Sensitivity was calculated as the proportion of true positives (number of samples from patients with previously diagnosed recurrent angioedema due to C1INH deficiency that were correctly identified as being from patients with recurrent angioedema due to C1INH deficiency by each test), and specificity was calculated as the proportion of true negatives (number of samples from healthy subjects that were correctly identified as being from subjects without recurrent angioedema due to C1INH deficiency by each test).

## Results

### Study population

The fC1INH LC-MS/MS assay from DBS was tested in 30 samples from patients (11 male and 19 female patients) with previously diagnosed recurrent angioedema due to C1INH deficiency and 100 samples (male/female ratio ∼50%/50%) from healthy subjects (Table I). Some of the samples have been analyzed for a previous publication and reanalyzed for the present study.^27^ One of the samples had insufficient quantity to be tested on all 3 assays. All patients had more than 20 years of follow-up in the allergy practice, had a documented angioedema attack within 5 years before sample collection, and were receiving therapies for HAE treatment. All patients had a previous diagnosis of recurrent angioedema due to C1INH deficiency (29 patients with HAE-C1INH and 1 patient with acquired angioedema due to C1INH deficiency, established by biochemical testing [antigenic C4 and/or antigenic C1INH and/or fC1INH] and family history, with genetic tests for *SERPING1* obtained in case of any ambiguity) as a part of their regular care before and unrelated with the sample collection for this study. The fC1INH ELISA and fC1INH chromogenic assays were tested in a subset of samples (29 samples from patients [11 male and 18 female patients] with previously diagnosed recurrent angioedema due to C1INH deficiency [28 patients with HAE-C1INH and 1 patient with acquired angioedema due to C1INH deficiency] and 50 samples from healthy subjects [20 male and 30 female subjects]; Table I).

**Table I:** Sample subsets used for the analyses

Subset	Healthy controls		Patients with recurrent angioedema due to C1INH deficiency
Sample subset	A	B	C
n	50	50	29
fC1INH LC-MS/MS assay from DBS (DBS main analysis)	✓	✓	✓∗
fC1INH ELISA		✓	✓
fC1INH chromogenic assay		✓	✓
fC1INH LC-MS/MS assay from DBS (DBS subset analysis)		✓	✓

∗ DBS main analysis included 1 additional patient with HAE-C1INH in subset C (30 patients with previously diagnosed angioedema due to C1INH deficiency were included in this analysis).

### Comparison of fC1INH assays

The fC1INH LC-MS/MS assay from DBS demonstrated similar sensitivity and specificity in identifying patients with recurrent angioedema due to C1INH deficiency when contrasted with the currently available fC1INH chromogenic assay (Table II). When contrasted with the fC1INH LC-MS/MS assay from DBS and the fC1INH chromogenic assay, fC1INH ELISA sensitivity was lower in the ELISA main analysis and similar in the ELISA alternative analysis; fC1INH ELISA specificity was similar to that of the other 2 assays in both the ELISA main analysis and the ELISA alternative analysis (Table II). Furthermore, the sensitivity and specificity of the fC1INH LC-MS/MS assay from DBS were similar in the full analysis set and in the subset of samples that was also reanalyzed using the fC1INH ELISA and chromogenic assays (Table II).

**Table II:** Comparison of fC1INH assays

Characteristic	fC1INH LC-MS/MS DBS assay (DBS main analysis)	fC1INH LC-MS/MS DBS assay (DBS subset analysis)	fC1INH ELISA (ELISA main analysis)	fC1INH ELISA (ELISA alternative analysis)∗	fC1INH chromogenic assay
N_AE_	30 (11 male, 19 female)	29 (11 male, 18 female)	29 (11 male, 18 female)	29 (11 male, 18 female)	29 (11 male, 18 female)
N_HS_	100 (male/female ratio ∼ 50%/50%)	50 (20 male, 30 female)	50 (20 male, 30 female)	50 (20 male, 30 female)	50 (20 male, 30 female)
AUC of the ROC curve (95% CI)	0.996 (0.989-1.000)	0.997 (0.989-1.000)	1.00	1.00	1.00
Threshold	359 mU/mL	431 mU/mL	41% of the normal	78% of the normal	119% of the normal
Positives, n/(N_AE_ + N_HS_)	31/130	32/79	18/79	30/79	28/79
Negatives, n/(N_AE_ + N_HS_)	99/130	47/79	61/79	49/79	51/79
True positives, n/N_AE_	28/30	29/29	18/29	29/29	28/29
Sensitivity	0.93	1.00	0.62	1.00	0.97
Negative predictive value	0.98	1.00	0.82	1.00	0.98
True negatives, n/N_HS_	97/100	47/50	50/50	49/50	50/50
Specificity	0.97	0.94	1.00	0.98	1.00
Positive predictive value	0.90	0.91	1.00	0.97	1.00

*N*_*AE*_, Number of patients with previously diagnosed recurrent angioedema due to C1INH deficiency; *N*_*HS*_, number of healthy subjects.

∗ In the ELISA alternative analysis, 10 samples from patients with confirmed HAE with ELISA results in the equivocal zone (41%-67% of the normal) and 1 sample with ELISA results negative for HAE (>67%) as per manufacturer’s instructions were below healthy control mean − 1.96 SD cutoff and were therefore evaluated as positive.

## Discussion

On the basis of the clinical presentation, patients with HAE may be misdiagnosed with other types of angioedema (allergic or nonallergic) and/or gastrointestinal conditions.^36^ In patients with HAE, receiving a correct diagnosis is essential to support appropriate management of the disease and avoid ineffective treatments (eg, antihistamines, epinephrine, and corticosteroids)^37^ and/or unnecessary interventions (eg, appendectomies).^38^ Most importantly, timely HAE diagnosis may help to reduce mortality due to laryngeal attacks, considering that previous studies showed that up to 9 in 10 deaths due to asphyxiation in patients with HAE occurred in undiagnosed patients.^8^^,^^39^ Furthermore, it is important to distinguish HAE-C1INH from clinically similarly presenting acquired angioedema conditions (eg, acquired angioedema due to C1INH deficiency, acquired angiotensin-converting enzyme inhibitor–associated angioedema, idiopathic histaminergic angioedema, and idiopathic nonhistaminergic angioedema) to enable family screening of patients with HAE as recommended by international guidelines.^9^^,^^11^

Several unmet needs in the laboratory diagnostics of HAE persist. fC1INH is an essential diagnostic biomarker for HAE-C1INH, and the potential of fC1INH as a prognostic biomarker to monitor disease activity has also been discussed.^15^ However, currently available fC1INH ELISA and fC1INH chromogenic assays require technical expertise and laboratory equipment,^13^ which may limit their accessibility in underserved areas. For example, it has been suggested that more than 95% of patients with HAE from China and India may remain undiagnosed, with availability of diagnostic facilities only in a select few centers as well as challenges in performing fC1INH assays that require careful sampling handling and technical expertise cited among key reasons for this rate of underdiagnosis.^23^^,^^24^ Diagnosis of HAE Type II is particularly impeded by a lack of fC1INH testing, even when other diagnostic tests for HAE (antigenic C4 and/or antigenic C1INH testing) are available, because antigenic C1INH and antigenic C4 testing may fail to correctly identify patients with HAE Type II.^24^^,^^25^ Of the 3 guideline-recommended diagnostic tests, fC1INH is always reduced in patients with HAE Type II, whereas antigenic C1INH is normal or elevated, and C4 may also be normal in these patients.^3^^,^^14^ Conventional fC1INH assays measure C1INH function through C1s protease activity, although C1s protease is not considered to be involved in the pathology of HAE.^40^ Alternative assays have also been proposed for fC1INH measurement through the protease activity of activated factor XII or plasma kallikrein, both of which are involved in bradykinin production and, consecutively, swelling attacks in HAE.^16^^,^^40^ Targeted Sanger sequencing has been suggested as an alternative for family screening of HAE in developing countries because of cost-effectiveness and easier sample handling versus performing all of the HAE biomarker tests (C4, C1INH, and fC1INH).^41^ However, some studies have shown low diagnostic sensitivity of Sanger sequencing, with sequencing failing to correctly identify a notable proportion of patients with biochemically proven C1INH deficiency as having HAE-C1INH, and the specificity of Sanger sequencing to establish HAE diagnosis may vary.^24^ Therefore, options for validated diagnostic assays that are widely accessible are still needed to improve diagnostics of HAE-C1INH worldwide.

Results of our study show similar sensitivity of the fC1INH LC-MS/MS assay from DBS to identify patients with recurrent angioedema due to C1INH deficiency when contrasted with the fC1INH chromogenic assay, and similar specificity of all 3 fC1INH assays. Meanwhile, the fC1INH ELISA had lower sensitivity when contrasted with either the fC1INH LC-MS/MS assay from DBS or the fC1INH chromogenic assay. These results supplement the results of previous studies that compared fC1INH ELISA and chromogenic assays and found similar results overall,^18^^,^^42^^,^^43^ with one study reporting some false positives with the fC1INH chromogenic assay,^42^ and another reporting low negative predictive value and, therefore, low sensitivity with the fC1INH ELISA.^44^ The fC1INH ELISA was reported to perform better when the reference ranges adjusted in the specific laboratory are used instead of manufacturer-recommended guidelines.^14^^,^^44^

Some of these older studies classified fC1INH ELISA results as either positive or negative^14^^,^^42^^,^^44^; however, other more recent studies reported following manufacturer’s classification of fC1INH ELISA results as normal, equivocal, or low.^18^^,^^40^ Equivocal results from the commercial fC1INH ELISAs in these studies were relatively frequent (7 of 31 [or 22.6%] enrolled patients with HAE^18^ and 38 of 87 [or 43.7%] enrolled patients with HAE,^40^ respectively) and similar to our study, in which the fC1INH ELISA had equivocal results in 10 of 29 (34.5%) patients with previously diagnosed recurrent angioedema due to C1INH deficiency. It is recommended to repeat equivocal ELISA results for confirmation, or, preferably, repeat the fC1INH testing using a chromogenic assay; however, this may not be practical in resource-constrained settings.^18^ The fC1INH chromogenic assay has categories only for normal and low fC1INH levels, bypassing the potential ambiguity of equivocal fC1INH ELISA results.^18^ However, the fC1INH chromogenic assay has been shown to return incorrect results when performed from samples handled under suboptimal conditions likely to be encountered in day-to-day laboratory work, highlighting the importance of handling and shipping conditions for this assay.^19^ The fC1INH ELISA also requires immediate centrifugation and refrigeration or freezing of the sample before the administration of the assay.^13^ Incorrect storage or shipping conditions are likely to cause sample decay, which correspondingly may lead to false-positive HAE diagnoses.^19^^,^^45^ Nevertheless, current clinical guidelines consider biochemical C1INH testing to be effective, with *SERPING1* sequencing identified as potentially supportive in the diagnostic workup of some patients with HAE-C1INH, for example, for prenatal testing or when the biochemical testing results are ambiguous.^3^ In contrast with blood samples for the fC1INH ELISA and chromogenic assays, fC1INH in DBS has been shown to be stable for up to 134 days at room temperature and up to 3 days at 45°C.^27^ Because the fC1INH LC-MS/MS assay from DBS does not require refrigeration and specific storage conditions, its application may prove beneficial when optimal handling and storage conditions cannot be assured, for example, in health care facilities from resource-constrained settings. Furthermore, straightforward evaluation with no equivocal values and robust results regardless of handling conditions with the fC1INH LC-MS/MS assay from DBS may reduce the need for retesting and contribute to simplification of the diagnostic algorithm for HAE-C1INH.

Another potential advantage for the fC1INH-DBS LC-MS/MS assay in resource-constrained settings is that blood sampling for DBS can be collected from capillary blood, unlike the fC1INH ELISA and fC1INH chromogenic assays, which require venipuncture for blood sampling. In addition to being less invasive, with lower patient discomfort and a smaller volume of blood collected, sampling of capillary blood is less complicated than sampling of venous blood, requires minimal equipment, and may not require staff with phlebotomy expertise.^28^, ^29^, ^30^ Increasing the availability of HAE diagnostics is one of the steps required to reduce the disparity of HAE management worldwide; however, raising physician awareness of this disease and ensuring access to effective HAE medications are also necessary to achieve equitable HAE management.^21^^,^^24^

Case-control design, in which a test is separately evaluated in patients with known disease and in healthy subjects, is a known limitation of study design for diagnostic test evaluation studies, because this approach has been reported to overestimate the diagnostic accuracy of the test compared with the diagnostic test evaluation studies that investigate clinical populations.^46^ However, our study used 2 different alternative tests to contrast with the fC1INH LC-MS/MS assay from DBS, and the same alternative tests were used to evaluate both positive and negative results. Furthermore, this study was conducted in the United States under optimal sample handling conditions as defined by a clear protocol. These conditions are unlikely to be consistently achieved in the real world, which further limits the interpretation of sensitivity and specificity for the fC1INH ELISA and fC1INH chromogenic assay.

### Conclusion

The sensitivity and specificity for identifying patients with recurrent angioedema due to C1INH deficiency were similar between the fC1INH LC-MS/MS assay from DBS and the fC1INH chromogenic assay. fC1INH ELISA results were only similar to those of other assays in the ELISA alternative analysis wherein equivocal ELISA results were treated as positive. In the ELISA main analysis wherein equivocal fC1INH ELISA results were not treated as positive, the fC1INH ELISA had notably lower sensitivity when contrasted with that of the fC1INH LC-MS/MS assay from DBS and the fC1INH chromogenic assay. The results from the ELISA main analysis are more reflective of real-world clinical practice in which equivocal fC1INH ELISA results are expected to be repeated or retested using the fC1INH chromogenic assay versus the results from the ELISA alternative analysis. The fC1INH LC-MS/MS assay from DBS achieves similar results to existing measurement methods without requiring venous blood collection, sample centrifugation, and sample refrigeration. With its simpler methodology and comparable results, the fC1INH LC-MS/MS assay from DBS may allow simplification of the existing diagnostic algorithm and more widespread testing for HAE, especially in underserved areas of the world.

**Key messages**

• **fC1INH is a key biomarker for diagnosis of HAE due to C1INH deficiency.**
• **We assessed sensitivity and specificity of fC1INH DBS, ELISA, and chromogenic assays.**
• **fC1INH DBS assay had simpler methodology and similar performance to conventional ELISA and chromogenic assays.**

## Disclosure statement

This study was sponsored by Takeda Development Center Americas, Inc. Takeda Development Center Americas, Inc, also provided funding to Excel Scientific Solutions, Inc, for support in writing and editing this article.

Disclosure of potential conflict of interest: J. A. Bernstein has received research grants from BioCryst, CSL Behring, Ionis, KalVista, Pharming, and Takeda; served on speakers bureaus/received honoraria from BioCryst, CSL Behring, Pharming, and Takeda; served as a consultant/on advisory boards for Astria, BioCryst, BioMarin, CSL Behring, Cycle Pharmaceuticals, Ionis, KalVista, Pharming, Pharvaris, ONO, and Takeda; is an advisory board member of the US Hereditary Angioedema Association; and is president of the 10.13039/100002567American Academy of Allergy, Asthma & Immunology. J. Cheng, T. Pisani, R. E. Whitaker, D. Nova Estepan, and N. Inhaber are employees of Takeda Development Center Americas, Inc; and hold stock/stock options in Takeda Pharmaceutical Company Limited. D. Sexton was an employee of Takeda Development Center Americas, Inc, at the time the study was conducted, and holds stock/stock options in Takeda Pharmaceutical Company Limited.
